# Effectiveness of the 23-valent pneumococcal polysaccharide vaccine against invasive pneumococcal disease among 948,263 individuals ≥ 65 years of age: a Danish cohort study

**DOI:** 10.1007/s10096-022-04513-5

**Published:** 2022-10-25

**Authors:** Katrine Finderup Nielsen, Lise Birk Nielsen, Frederikke Kristensen Lomholt, Sarah Kristine Nørgaard, Hans-Christian Slotved, Tine Dalby, Kurt Fuursted, Charlotte Sværke Jørgensen, Palle Valentiner-Branth

**Affiliations:** 1 Department of Infectious Disease Epidemiology and Prevention, Statens Serum Institut, Copenhagen, Denmark; 2 Department of Bacteria, Parasites and Fungi, Statens Serum Institut, Copenhagen, Denmark; 3 Department of Microbiological Special Diagnostics, Statens Serum Institut, Copenhagen, Denmark

**Keywords:** Pneumococcal polysaccharide vaccine, Vaccine effectiveness, Invasive pneumococcal disease, Epidemiology

## Abstract

This study aimed to estimate the effectiveness of the 23-valent pneumococcal polysaccharide vaccine (PPV23) against invasive pneumococcal disease (IPD) among individuals ≥ 65 years of age. We used Danish nationwide databases to obtain information on PPV23 vaccination, covariates, and IPD and linked data on an individual level using a unique personal identifier. A total of 948,263 individuals were included and followed between June 15, 2020, and September 18, 2021 (58.6% were vaccinated during follow-up). The adjusted vaccine effectiveness was 42% (95% confidence interval (CI): 9–63%) for all-serotype IPD and 58% (95% CI: 21–78%) for PPV23-serotype IPD, using no vaccination as the reference.

**Supplementary information:**

The online version contains supplementary material available at 10.1007/s10096-022-04513-5.

## Introduction

Infection with *Streptococcus pneumoniae* can cause invasive pneumococcal disease (IPD), a severe and life-threatening condition [1]. On April 22, 2020, the Danish government initiated a free-of-charge pneumococcal vaccination programme with a 23-valent pneumococcal polysaccharide vaccine (PPV23) [2]. The programme initially targeted individuals at particularly high risk of IPD (e.g. individuals with chronic illness, dysfunctional spleen or immunodeficiency), and on June 15, 2020, the vaccination programme was expanded to include all individuals ≥ 65 years old [2]. Vaccine effectiveness and antibodies to PPV23-serotypes have been seen to wane over time [3–6]. Hence, current Danish guidelines recommend repeated vaccination every 6 years for those at increased risk of IPD [7].

The aim of this study was to estimate the effectiveness of PPV23 against all-serotype IPD and PPV23-serotype IPD in individuals ≥ 65 years, following the introduction of the Danish national PPV23 vaccination programme for this group.

## Methods

In Denmark, all residents are assigned a unique 10-digit civil personal registration number (CPR number), enabling the linkage of different nationwide Danish registries on an individual level [8]. The Danish Vaccination Register (DVR) holds data on vaccinations administered to individuals, including both privately purchased and free-of-charge vaccinations [9]. Since November 15, 2015, all vaccinators have been obliged to register vaccines in this registry. From the DVR, information on vaccination with PPV23, the 7- and 13-valent pneumococcal conjugate vaccines (PCV7 and PCV13), and influenza vaccines was obtained by using Anatomical Therapeutic Chemical (ATC) classification codes (Table S2). Information on IPD, defined as the detection of *S. pneumoniae* from blood, cerebrospinal fluid, or other normally sterile sites, were obtained from the Danish Microbiology Database (MiBa) [10]. This national database contains real-time information on all microbiological laboratory test results from all hospital-associated departments of clinical microbiology in Denmark. Hence, the data covers all tests for IPD in Denmark. The Danish National Patient Registry (DNPR) holds information on all admissions to non-psychiatric hospitals since 1977, and contacts to outpatient clinics and emergency departments since 1995 [8]. One primary and one or more optional secondary diagnoses are provided for each hospital-patient contact and coded according to the International Classification of Diseases, 10th revision (ICD-10) [8]. From DNPR, we obtained information on comorbid conditions in the five years prior to the date of study entry and computed a comorbidity score for each individual by use of the Charlson comorbidity index: 0 (low), 1–2 (moderate), and ≥ 3 (high) [11]. The Danish Civil Registration System (CPR) holds information on the date of birth, sex, migration, and date of death of all Danish residents [8].

**Fig. 1:**
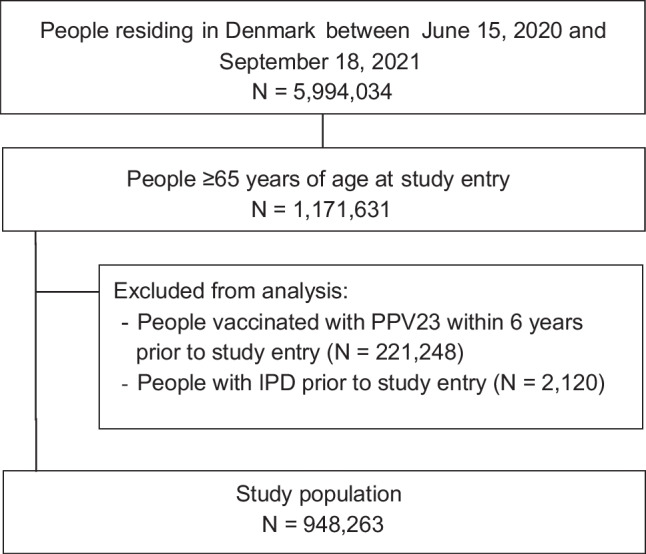
Flowchart of the selection of individuals included in the study population

All individuals ≥ 65 years, who were residing in Denmark between June 15, 2020, and September 18, 2021, were identified from the CPR. Since PPV23 is recommended every 6th year, individuals vaccinated with PPV23 within 6 years prior to the date of study entry were excluded, to assess the vaccine effectiveness within the vaccination programme. Furthermore, individuals with IPD before study entry were excluded as they might have unknown, underlying conditions, which can increase the risk of subsequent IPD [12]. The study population comprised of the remaining ≥ 65 year olds residing in Denmark. Figure 1 shows the derivation of the analysis sample. A sensitivity analysis was performed including those vaccinated with PPV23 within 6 years prior to study entry. In this analysis, all individuals not vaccinated within the study period were categorised as unvaccinated, independent of when they might have been vaccinated before the study began.

The study population was characterised according to sex, age, prior receipt of PCV7, PCV13, and influenza vaccine, comorbidity score, and selected individual comorbidities (myocardial infarction, chronic pulmonary disease, diabetes, renal disease, cancer, and congestive heart failure). All individuals were followed from June 15, 2020, or the date of immigration (index date), and until the date of IPD, emigration, death, or September 18, 2021, whichever came first. We considered exposure to PPV23 as a time-varying variable, meaning that individuals could move from the unvaccinated group to the vaccinated group, but not in the opposite direction, and contribute with risk time to the relevant group. Since an immune response to PPV23 usually occurs between 2 and 3 weeks after vaccination [13, 14], a lag period of 14 days is used when monitoring vaccine failure against IPD in Denmark. We used the same lag in this study, meaning that any outcomes occurring between 0 and 14 days following PPV23 vaccination were included in the unvaccinated group. We used Cox proportional hazards regression models to estimate hazard ratios (HRs) with calendar time as the underlying timescale and adjusted for sex and age as categorical variables, along with 95% confidence intervals (CIs), comparing PPV23 vaccination with no vaccination. The vaccine effectiveness (VE) for all-serotype IPD and PPV23-serotype IPD was calculated as (1 − HR)*100%. The statistical analyses were conducted using SAS software version 9.4 (Cary, NC, USA).

## Results

A total of 1,171,631 individuals ≥ 65 years were residing in Denmark between June 15, 2020, and September 18, 2021, and after the exclusion, 948,263 individuals were included in the study population (Fig. 1). Among these, 555,300 (58.6%) were vaccinated with PPV23 during follow-up. All covariates were distributed equally among unvaccinated and vaccinated individuals except that people vaccinated with PPV23 were more likely to have received an influenza vaccine within two years prior to study entry (Table 1). The median follow-up time for all-serotype IPD was 210 days (IQR: 90–336). The adjusted VE estimates was 42% (95% CI: 9–63%) for all-serotype IPD, and 58% (95% CI: 21–78%) for PPV23-serotype IPD (Table 2). Including the 221,248 individuals vaccinated with PPV23 within 6 years prior to study entry increased the VE point estimates, but not significantly (Table S1).

**Table 1:** Patient characteristics according to vaccination status

	Study population at entry, *n* (%)	Vaccinated with PPV23 during the study period, *n* (%)
Total	948,263 (100)	555,300 (100)
Female sex	511,869 (54.0)	301,892 (54.4)
Age, years		
Median [IQR]	74 [69–79]	74 [69–79]
< 75	545,298 (57.5)	320,551 (57.7)
75–84	313,782 (33.1)	190,882 (34.4)
≥ 85	89,183 (9.4)	43,867 (7.9)
Receipt of PCV7 prior to study entry	< 5	< 5
Receipt of PCV13 prior to study entry	16,763 (1.8)	12,227 (2.2)
Receipt of influenza vaccine within 2 years prior to study entry	468,748 (49.4)	360,803 (65.0)
Charlson comorbidity index score*		
0	706,171 (74.5)	411,921 (74.2)
1–2	201,731 (21.3)	120,454 (21.7)
≥ 3	40,361 (4.3)	22,925 (4.1)
Individual comorbidities within 5 years prior to study entry		
Myocardial infarction	13,931 (1.5)	8,087 (1.5)
Chronic pulmonary disease	23,327 (2.5)	13,889 (2.5)
Diabetes mellitus	21,465 (2.3)	12,031 (2.2)
Cancer	96,205 (10.2)	59,415 (10.7)
Congestive heart failure	16,098 (1.7)	8,844 (1.6)

Persons vaccinated with PPV23 during follow-up contributed to the analysis for both unvaccinated and vaccinated as appropriate. *Within 5 years prior to study entry. Score of 0 = low comorbidity, score of 1–2 = moderate comorbidity, score of ≥ 3 = high comorbidity. Abbreviations*: IQR*, interquartile range; *PCV7*, 7-valent pneumococcal conjugate vaccine; *PCV13*, 13-valent pneumococcal conjugate vaccine; *PPV23*, 23-valent polysaccharide pneumococcal vaccine

**Table 2:** Hazard ratios and vaccine effectiveness against all-serotype IPD and PPV23-serotype IPD, comparing vaccination with PPV23 with no vaccination, June 15, 2020, to September 18, 2021

				Hazard ratio (95% CI)		VE, % (95% CI)	
Outcomes	Vaccination status	Events, *n*	PYRS	Unadjusted	Adjusted*	Unadjusted	Adjusted*
All-serotype IPD	Unvaccinated	66	656,685	1 (reference)	1 (reference)	1 (reference)	1 (reference)
	Vaccinated	33	512,301	0.57 (0.36 to 0.89)	0.58 (0.37 to 0.91)	43 (11 to 64)	42 (9 to 63)
PPV23-serotype IPD	Unvaccinated	43	656,685	1 (reference)	1 (reference)	1 (reference)	1 (reference)
	Vaccinated	14	512,301	0.40 (0.21 to 0.77)	0.42 (0.22 to 0.79)	60 (23 to 79)	58 (21 to 78)

^*^Adjusted for age and sex as categorical variables. Abbreviations*: CI*, confidence interval; *IPD*, invasive pneumococcal disease; *PPV23*, 23-valent polysaccharide pneumococcal vaccine; *PYRS*, person years

## Discussion

In this Danish nationwide cohort study comprising individuals ≥ 65 years, vaccination with PPV23 was associated with a significantly lower risk of all-serotype IPD and PPV23-serotype IPD relative to no vaccination. To our knowledge, this is the first study to examine the effectiveness of PPV23 vaccination against IPD after the initiation of the Danish national vaccination programme for individuals ≥ 65 years old. Previous reviews reported pooled VE estimates against all-serotype IPD in ≥ 60 year olds ranging from 45% (95% CI: 15–65%) to 59% (95% CI: 35–74%) in the included observational studies [6, 15], and our results fall within this range. More recently, in those ≥ 60 years of age, one systematic review covering six observational studies reports VE estimates against PPV23-serotype IPD ranging from 27% (95% CI: 17–35%) to 42% (95% CI: -2 to 67%) [16]. Another review with 12 included observational studies, reported VE estimates ranging from 25% (95% CI: 11–37%) to 72.8% (95% CI: 59.1–81.8%) [17]. In accordance, we also found a statistically significant lower risk within this range of PPV23-serotype IPD following vaccination with PPV23 compared with no vaccination.

Many factors may influence the different VE estimates between the referenced studies and our studies, such as the health and age of the study populations, differences in dominant serotypes in the countries where the studies were conducted, waning protection, and length of follow-up. E.g., our study population of 948,263 individuals is larger than in the referenced studies, and our study design used a lag of 14 days after vaccination to avoid incorrectly decreasing the VE, which was not applied in the referenced studies. However, our follow-up time of 1 year and 3 months was relatively short compared to between 1 and 14 years in the mentioned reviews. In 2020, serotypes 8, 3, and 22F were the most predominant serotypes causing IPD in those aged ≥ 65 in Denmark [18].

A major strength of our study is the nationwide, cohort design, covering all individuals ≥ 65 years residing in Denmark during the study and with complete information on follow-up. Due to the mandatory registration of vaccinations in the DVR, misclassification of information on PPV23 is believed to be unlikely, and if any, it is not expected to be related to later outcomes. The study period covers a time when the COVID-19 pandemic was ever-present with non-pharmacological interventions curbing the transmission of SARS-CoV-2, leading to a decline in IPD cases across the world, including in Denmark [19]. Accordingly, we observed a low number of IPD cases during the study period despite the size of the study population, resulting in wide CIs and halting the inclusion of further covariates into the Cox regression model or subgroup analyses.

We chose to exclude all individuals with PPV23 vaccination within 6 years of study entry to assess VE in a presumably more homogenous group, i.e., those who received PPV23 as part of the vaccination programme for all individuals ≥ 65 years. Prior to June 15, 2020, only the most vulnerable were offered PPV23. However, a sensitivity analysis shows that excluding them does not impact the final VE estimates significantly. This might be due to the opposite effects including this population could have on the VE: including those more vulnerable to IPS increases the risk of IPS in the unvaccinated group, and thereby the VE estimates; while previous vaccination increases the immunity among those unvaccinated, and thereby lowers the risk of IPS and the VE estimates. The similar VE in the two analyses could indicate a waning of effect from PPV23 vaccination since the VE does not improve with previous vaccination. Furthermore, it cannot be ruled out that health-seeking behaviour differed between the unvaccinated and vaccinated groups, indicated by the larger proportion of influenza-vaccinated individuals in the PPV23-vaccinated group. However, the similar profiles of the group’s receipt of the different pneumococcal vaccines, as well as diagnosed comorbidities, seem to contradict this. Lastly, due to the observational design of the present study, any causal relationship cannot be assessed.

In conclusion, our study shows that vaccination with PPV23 is associated with moderate protection against all-serotype IPD and PPV23-serotype IPD in individuals ≥ 65 years, although the estimates of the association had limited statistical precision. These findings provide evidence supporting a continuation of the Danish national PPV23 programme.

## Supplementary information

Below is the link to the electronic supplementary material.

Supplementary file1 (DOCX 18 KB)

## Data Availability

The data included in this research is part of the Danish national vaccination surveillance system at Statens Serum Institut. The data is available for research upon reasonable request and with permission from the Danish Data Protection Agency and the Danish Health and Medicines Authority.

## Materials availability

The data included in this research is part of the Danish national vaccination surveillance system at Statens Serum Institut. The data is available for research upon reasonable request and with permission from the Danish Data Protection Agency and the Danish Health and Medicines Authority.
