# Supplementary material for: Effectiveness of the 23-valent pneumococcal polysaccharide vaccine against invasive pneumococcal disease among 948,263 individuals ≥ 65 years of age: a Danish cohort study
Source: Eur J Clin Microbiol Infect Dis. 2022 Oct 25;41(12):1473–7. doi: 10.1007/s10096-022-04513-5 (PMC9593971; doi:10.1007/s10096-022-04513-5)
Supplement: Supplementary file 1 — Supplementary file1 (DOCX 18 KB) [file 10096_2022_4513_MOESM1_ESM.docx]

# Supplementary material

| **Table S1. Hazard ratios and vaccine effectiveness against all-serotype IPD and PPV23-serotype IPD including individuals with PPV23 vaccination within 6 years, comparing vaccination with PPV23 with no vaccination, June 15 2020 to September 18 2021** | | | | | | | |
| --- | --- | --- | --- | --- | --- | --- | --- |
|  |  |  |  | **Hazard ratio (95% CI)** | | **VE, % (95% CI)** | |
| **Outcomes** | **Vaccination status** | **Events, n** | **PYRS** | **Unadjusted** | **Adjusted*** | **Unadjusted** | **Adjusted*** |
| All-serotype IPD | Unvaccinated | 114 | 915,407 | 1 (reference) | 1 (reference) | 1 (reference) | 1 (reference) |
|  | Vaccinated | 34 | 516,659 | 0.47 (0.32 to 0.70) | 0.48 (0.32 to 0.72) | 53 (30 to 68) | 52 (28 to 68) |
|  |  |  |  |  |  |  |  |
| PPV23-serotype IPD | Unvaccinated | 69 | 915,407 | 1 (reference) | 1 (reference) | 1 (reference) | 1 (reference) |
|  | Vaccinated | 14 | 516,659 | 0.35 (0.19 to 0.63) | 0.36 (0.20 to 0.65) | 65 (37 to 81) | 64 (35 to 80) |

*Adjusted for age and sex as categorical variables.

Abbreviations*:* CI, confidence interval; IPD, invasive pneumococcal disease; PPV23, 23-valent polysaccharide pneumococcal vaccine; PYRS, person years.

| **Table S2. ATC codes used for data extraction** | | | |
| --- | --- | --- | --- |
| **Vaccinetype** | **ATC-code** |  | |
| PCV7 | J07AL02 | |  |
| PCV13 | J07AL02 | |  |
| PPV23 | J07AL01 | |  |
| Influenza | J07BB01, J07BB02 | |  |
| Abbreviations*:* ATC, Anatomical Therapeutic Chemical; PCV7, 7-valent pneumococcal conjugate vaccine; PCV13, 13-valent pneumococcal conjugate vaccine; PPV23, 23-valent polysaccharide pneumococcal vaccine | | | |
